# Supplementary material for: Health providers readiness in managing intimate partner violence in public health institutions, Ethiopia
Source: PLoS One. 2023 Dec 22;18(12):e0295494. doi: 10.1371/journal.pone.0295494 (PMC10745191; doi:10.1371/journal.pone.0295494)
Supplement: S1 File — (DOCX) [file pone.0295494.s001.docx]

**Assumptions and the model fitness t-test and plot**

| **Model Summary** | | | | | | | | | |
| --- | --- | --- | --- | --- | --- | --- | --- | --- | --- |
| Model | R | R Square | Adjusted R Square | Std. Error of the Estimate | Change Statistics | | | | |
|  |  |  |  |  | R Square Change | F Change | df1 | df2 | Sig. F Change |
| 1 | .827^a^ | .684 | .672 | .28602 | .684 | 55.086 | 16 | 407 | .000 |
|  | | | | | | | | | |


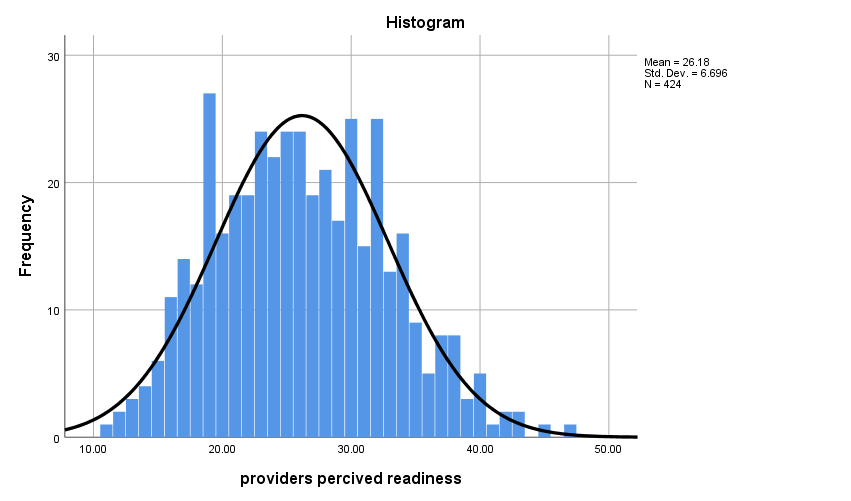


histogram /Q-Q plot was used to check the multivariate normality


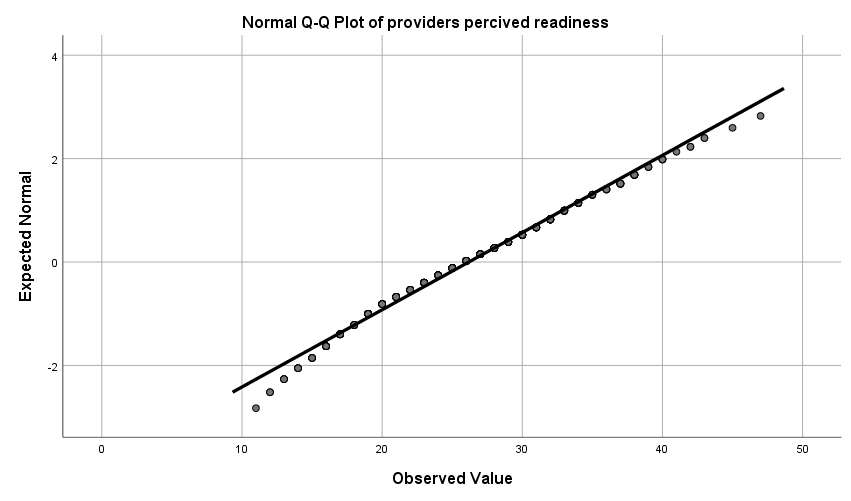


| **Tests of Normality** | | | | | | |
| --- | --- | --- | --- | --- | --- | --- |
|  | Kolmogorov-Smirnov^a^ | | | Shapiro-Wilk | | |
|  | Statistic | df | Sig. | Statistic | df | Sig. |
| providers percived readiness | .055 | 424 | .003 | .989 | 424 | .003 |
| a. Lilliefors Significance Correction | | | | | | |

Multicollinearity was checked by examining the variance inflation factors (VIF), and it showed no multicollinearity on the final model: the value for each variable was less than ten

| **Coefficients^a^** | | | | | | | | |
| --- | --- | --- | --- | --- | --- | --- | --- | --- |
| Model | | Unstandardized Coefficients | | Standardized Coefficients | t | Sig. | Collinearity Statistics | |
|  |  | B | Std. Error | Beta |  |  | Tolerance | VIF |
| 1 | (Constant) | 16.783 | 2.105 |  | 7.972 | .000 |  |  |
|  | percivedknowledge | .210 | .048 | .206 | 4.386 | .000 | .995 | 1.005 |
|  | tr | -2.023 | .877 | -.108 | -2.308 | .021 | .999 | 1.001 |
|  | Attitiude | .172 | .051 | .157 | 3.344 | .001 | .995 | 1.005 |
| a. Dependent Variable: providers percived readiness | | | | | | | | |

homoscedasticity (constant variance), was checked and which showed that the residuals were similar around the regression line
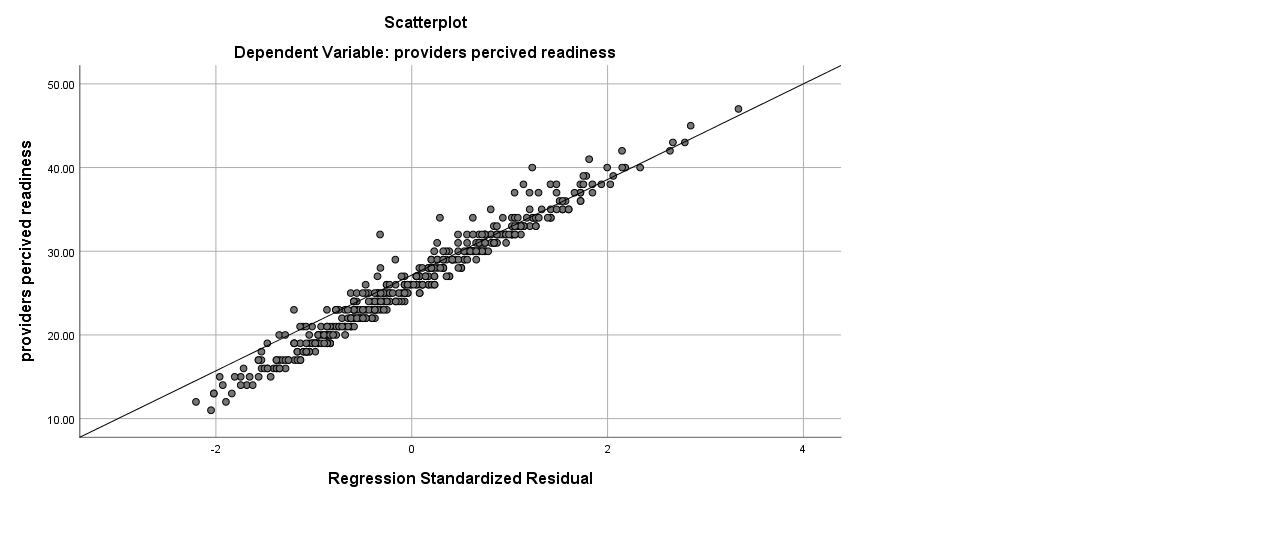


| **Correlations** | | | | |
| --- | --- | --- | --- | --- |
|  | | percivedknowledge | tr | Attitiude |
| percivedknowledge | Pearson Correlation | 1 | -.015 | -.065 |
|  | Sig. (2-tailed) |  | .761 | .182 |
|  | N | 424 | 424 | 424 |
| tr | Pearson Correlation | -.015 | 1 | -.029 |
|  | Sig. (2-tailed) | .761 |  | .556 |
|  | N | 424 | 424 | 424 |
| Attitiude | Pearson Correlation | -.065 | -.029 | 1 |
|  | Sig. (2-tailed) | .182 | .556 |  |
|  | N | 424 | 424 | 424 |
