# Supplementary material for: Health providers readiness in managing intimate partner violence in public health institutions, Ethiopia
Source: PLoS One. 2023 Dec 22;18(12):e0295494. doi: 10.1371/journal.pone.0295494 (PMC10745191; doi:10.1371/journal.pone.0295494)
Supplement: Attachment — Submitted filename: renamed_a0acc.docx [file pone.0295494.s004.docx]

Response to reviewers’ comment

We thank both reviewers for the interest they shown to review our paper and for their valuable comments. We made all modification based on the comment provided and the change made indicated in in line with each comment.

Review Comments to the Author

Reviewer #1: Intimate partner violence is a serious public health issue and provider knowledge and preparedness to care for patients experiencing IPV is an important part of the public health response to this issue. This manuscript presents data regarding the readiness of providers in Hawassa City, Ethiopia. The manuscript could be improved by addressing the following issues:

The sample size calculation is presented before any information about what data is being collected. Mean of what?? The manuscript should be reorganized so that the sample size calculation comes after the variables.

1. We used a **single population mean** formula to calculate our sample size. However, there was no similar study done to determine the variance. Then a pilot study was conducted to determine mean and variance of perceived readiness in managing IPV. on 10% of the source population.

Study variables: The PREMIS instrument is identified after the description of the study variables. The use of a standardized instrument is a strength of this study. The instrument identification and description should be at the start of the variable section.

Strength of Study and Limitations should not be bulletted lists.

2. We avoid the bullets

Overall, the manuscript needs to be edited for English language. There are sections that are hard to read and sentences that don't make sense.

3. We revised the language

Reviewer #2: This well-conducted research examines health provider’s readiness to address IPV in Ethiopia. This topic is clearly of relevance to PLOS-One.Formatting: OK.

Writing: Some grammatical errors (i.e. needed apostrophes), awkward or stilted language and odd phrasing (i.e. “needed to upsurge” on page 1 in the abstract. This submission need to be edited for English syntax.

Too many acronyms throughout (i.e. OPD & MCH). Better to write out names except for common acronyms such as IPV.

4. Uncommon acronyms removed

Title: Too complex. Perhaps just note Ethiopia (rather than city and state details)

and leave off the year.

5. The title modified to

Health providers readiness in managing intimate partner violence in public health institutions, Ethiopia.

Abstract: Clear and detailed. However, it is unusual to include such details as the p-value for significance and confidence interviews. Keep such details to the body of the submission.

6. p-value and confidence interval is removed

Introduction: Some strongly worded statements that are questionable. Looking at only the first paragraph, stating that IPV is the “most prevalent form of violence” is not usually stated as such. Also “It occurs without exclusion”… is an unusual way to make the point that it is common. “Almost all violent behavior in IPV psychological violence” – is not stated clearly. What does this mean? Globally, should be followed by a comma.

7. Revised

In the second paragraph, the point about homicides is appropriate but the subsequent connection to medical treatment and support does not follow.

8. Revised

In the third paragraph, “Health providers are front lines responsible” … This does not make sense.

9. Revised

Better to first introduce the global statistics and issues related to IPV. Then add the Ethiopian context in a separate section.

10. Revised

Research Methods: It is unusual to include a statistical formula regarding sample size. Simply delete and describe in the narrative.

11. The formula for sample size removed

The PREMIS tool is a strong choice. Was the full scale used or not? It is confusing to present the study variables separately if they are directly derived from the PREMIS (Which seems the case). Delete the section on study variables. Consolidate this information.

12. The tool has four sections

- perceived readiness (9 items)
- Perceived knowledge (12 items)
- Attitude section (13 items)
- Practice section (11 items)

The dependent variable in section is perceived readiness and we believed it is useful to descriptively present the other section of the tool

Results: Great sample size (N = 428). The data analysis is largely simply descriptive. This is OK but could be more interesting i.e. if there was a logical comparison such as by gender or health care practice area.

Discussion: The comparison of the results to other PREMIS studies, especially other African studies, is interesting and appropriate.

Summary: In summary, this Ethiopian study of IPV readiness with a large sample size using a well-respected tool, the PREMIS, has much to recommend it. However, as currently written it needs a thorough edit for English syntax.

13. Recommendation added

References: Correctly formatted although digital object identifiers (dois) could be used as well.

Specific Issues: (no page numbers or line numbers so difficult to pinpoint specific issues.

14. Reference revised and DOI number included

p. 1 Abstract Introduction. The phrase “against all women” is incorrect. IPV is a significant problem but not for all.

15. Modified

6. PLOS authors have the option to publish the peer review history of their article (what does this mean?). If published, this will include your full peer review and any attached files.
