# Supplementary material for: Health providers readiness in managing intimate partner violence in public health institutions, Ethiopia
Source: PLoS One. 2023 Dec 22;18(12):e0295494. doi: 10.1371/journal.pone.0295494 (PMC10745191; doi:10.1371/journal.pone.0295494)
Supplement: Attachment — Submitted filename: Response to reviewer 2.docx [file pone.0295494.s005.docx]

Response to editor

1. In the Methods section please include the informed consent statement to reflect whether "written or verbal" informed consent was obtained from all participants for inclusion in the study.

The Ethical consideration section modified which include the statement

“Before the questionnaire was administered clear information on the nature and importance of the study was offered to the participants and informed verbal consents was obtained.”

Reviewer #1: The authors have addressed some of the reviewer comments. The following issues remain:

Abstract: "The strength of association...." The last two sentences have information that are not typically in an abstract. They should be removed.

the last two parts of abstract part removed

Introduction: Ethiopia should be added to very end of the aim to make clear for all readers where this is.

Ethiopia is added

Sample size calculation: Did the sample size calculation account for clustering? Given that the sample size was stratified, clustering should be included in the calculation.

Sample size was calculated by using single population mean. After selecting health facilities by simple random sampling, the final sample is proportionally allocated for each facility. We did not use any stratification or clustering

Study Variables: The authors did not address the guidance from the reviewers regarding the structure of this section. The PREMIS instrument should be described before it's subscales are included.

Some point is added about tool

Data collection: The data collection section is not about data collection. It is about the instrument. The content of this section should be moved up above the text in the study variable section. The use of the PREMIS is a strength of this study.

the content moved

Information about PREMIS presented under heading measurement

Methods: There is no data collection information. This should be added.

Added

Reviewer #2:

This interesting research examines health provider’s readiness to address IPV in Ethiopia. This topic is clearly of relevance to PLOS-One. The authors have responded conscientiously to a number of the reviewers’ comments and suggestions; but not all. The authors did not respond to at all to several of my suggestions.
The resubmission was confusing. The actual revised document was found after the first submission was presented (or perhaps this was the revised document without track changes… It was not immediately clear what was the case. Perhaps this was a function of the journal submission process, but it did add extra time to conduct the review.

Further, the revised submission did not have lines on the side, so difficult to make specific suggestions.

Formatting: OK.

Writing: Still some English syntax problems (i.e., in the Abstract the sentence “Variable with the P- value of < 0.05 was considered a statistically…” needs editing i.e., “Variables with a p-value of <0.05 were…”. Also, as this is a commonly accepted p-value, no need to include this statement, especially not in an abstract.

removed and corrections made

The authors add a section to the end of the paper listing the acronyms used. However, this does not help the reader who must interpret a number of unusual acronyms throughout (i.e., OPD & MCH). As before, better to write out names except for common acronyms such as IPV.

OPD & MCH removed

Title: Better.

Abstract: Better

Introduction: Better.

Research Methods: As before, the PREMIS tool is a strong choice. It is confusing to present the study variables separately and first, if they are directly derived from the PREMIS (which seems the case). Present the PREMIS information first – then describe the subscales (which are the study variables) Consolidate this information.

Change made according to recommendation

Results: As before, this is a great sample size (N = 428). As before, the data analysis is largely simply descriptive. This is OK but could be more interesting i.e., if there was a logical comparison such as by gender or health care practice area. The authors have stuck with their original descriptive description of the demographics.

We included a section with the title Health providers readiness by socio demographic characters and added a table to indicate comparison by sociodemographic characteristics

Discussion: The comparison of the results to other PREMIS studies, especially other African studies, is interesting and appropriate.

Summary: In summary and, as before, this Ethiopian study of IPV readiness with a large sample size using a well-respected tool, the PREMIS, has much to recommend it. However, the authors have not commented or revised several key recommendations from this reviewer such as reorganizing Methods to more directly credit the PREMIS tool and compare PREMIS results based on a key demographic variable such as gender or health care practice area. These would make the study less basic and potentially of more interest.

All recommendation given is included

References: Correctly formatted.
