# Supplementary material for: Health providers readiness in managing intimate partner violence in public health institutions, Ethiopia
Source: PLoS One. 2023 Dec 22;18(12):e0295494. doi: 10.1371/journal.pone.0295494 (PMC10745191; doi:10.1371/journal.pone.0295494)
Supplement: Attachment — Submitted filename: Response to editor.docx [file pone.0295494.s007.docx]

Additional Editor Comments:

We are unable to open your Supporting Information file [ SPSS data Health providers readiness in managing IPV.sav ]. Please kindly revise as necessary and re-upload

- We have attached both as CSV and SPSS file format. I can attach the file in other format if you have any preference format type

As the author did the linear regression, did they check the normality and all other assumption of the model? If yes, provide how they did; histogram/density or other plots are suggested as a supplementary file to see the outcome's distribution. Report all the other assumptions assessed before fitting the model, such as correlation, interaction, and confounding. If the correlation, interaction, and confounding were found, how was the variable selected in the model?

Response: -

- The assumptions and the model fitness t-test and plot provided as supplementary file
- The assumption were checked for the model fit and reported in paragraph 2 page 13

After fitting the model, did the author check the fitness of the model?

Report and interpret the intercept, R Squared, and F value of the model.

- Response: - Adjusted R square revealed that 68.0% of the dependent variable could be explained by the independent variable.

Reporting guidelines STOBE is requested to use.

- Response - STOBE checklist provided as supplementary file

Response

All feedbacks incorporated in the method and result section. The following statement added in the method, data analysis section

- All variables having P-value ≤ 0.25 during the simple linear regression analysis and deemed important variable by the researcher were considered as candidates variables for the multivariable linear regression.

And the following two paragraphs added in the Factors associated with provider’s perceived readiness in managing IPV

Simple linear regression analysis was done to find the association of socio-demographic characteristics, health providers related and health facilities related factors on provider’s perceived readiness in managing IPV. During simple linear regression availability of protocol at practical area, receiving IPV training, perceived knowledge of providers in managing IPV and attitude towards IPV management showed statistically significant association with provider’s perceived readiness in managing IPV.

Eleven variables entered into the final multiple linear regression model. The final model has fulfilled all assumptions of normality and all the independent variables scored VIF less than five indicating non‐existence of multicollinearity. The model can explain 68% of the variation in provider’s perceived readiness. The overall regression model was good fit with F value of 3.48 and p value < 0.001. During the final model sex, marital status, profession, clinical work experience, practice and familiarity with institution’s policies regarding management of IPV were not found to be significant predictor.
