# Supplementary material for: Health providers readiness in managing intimate partner violence in public health institutions, Ethiopia
Source: PLoS One. 2023 Dec 22;18(12):e0295494. doi: 10.1371/journal.pone.0295494 (PMC10745191; doi:10.1371/journal.pone.0295494)
Supplement: Attachment — Submitted filename: Response to reviewers.docx [file pone.0295494.s008.docx]

**Response to reviewers**

**We acknowledge the reviewers for the constructive comments and their irreplaceable role they played in improving the paper**

**We responded for all comments provided (response provided are highlighted in bright green)**

**Comments to the Author**

1. If the authors have adequately addressed your comments raised in a previous round of review and you feel that this manuscript is now acceptable for publication, you may indicate that here to bypass the “Comments to the Author” section, enter your conflict-of-interest statement in the “Confidential to Editor” section, and submit your "Accept" recommendation.

Reviewer #1: All comments have been addressed

Reviewer #2: All comments have been addressed

2. Is the manuscript technically sound, and do the data support the conclusions?

The manuscript must describe a technically sound piece of scientific research with data that supports the conclusions. Experiments must have been conducted rigorously, with appropriate controls, replication, and sample sizes. The conclusions must be drawn appropriately based on the data presented.

Reviewer #1: Yes

Reviewer #2: Yes

3. Has the statistical analysis been performed appropriately and rigorously?

Reviewer #1: Yes

Reviewer #2: Yes

4. Have the authors made all data underlying the findings in their manuscript fully available?

The [PLOS Data policy](http://www.plosone.org/static/policies.action#sharing) requires authors to make all data underlying the findings described in their manuscript fully available without restriction, with rare exception (please refer to the Data Availability Statement in the manuscript PDF file). The data should be provided as part of the manuscript or its supporting information, or deposited to a public repository. For example, in addition to summary statistics, the data points behind means, medians and variance measures should be available. If there are restrictions on publicly sharing data—e.g. participant privacy or use of data from a third party—those must be specified.

Reviewer #1: Yes

Reviewer #2: Yes

5. Is the manuscript presented in an intelligible fashion and written in standard English?

PLOS ONE does not copyedit accepted manuscripts, so the language in submitted articles must be clear, correct, and unambiguous. Any typographical or grammatical errors should be corrected at revision, so please note any specific errors here.

Reviewer #1: Yes

Reviewer #2: Yes

6. Review Comments to the Author

Please use the space provided to explain your answers to the questions above. You may also include additional comments for the author, including concerns about dual publication, research ethics, or publication ethics. (Please upload your review as an attachment if it exceeds 20,000 characters)

Reviewer #1: The manuscript is much stronger. One final suggestion is to add to the study objective the regression analysis - add that you are also examining factors associated with providers' perceived readiness to manage IPV

**Response: objective related to regression analysis added in**

- **abstract section introduction last sentence**
- **page four line 70 and 72**

Reviewer #2: This interesting research examines health provider’s readiness to address IPV in Ethiopia. This topic is clearly of relevance to PLOS-One. The authors have responded conscientiously to a number of the reviewers’ comments and suggestions.
Formatting: OK.
Writing: Better
Title: Better.
Abstract: Better
Introduction: Better.
Research Methods: As before, the PREMIS tool is a strong choice. It is confusing to present the study variables separately and first if they are directly derived from the PREMIS (which seems the case). Present the PREMIS information first – then describe the subscales (which are the study variables) Consolidate this information.

**Response: based on recommendation the study variables section is consolidated to the measurement.**

Results: As before, this is a great sample size (N = 428).
Discussion: The comparison of the results to other PREMIS studies, especially other African studies, is interesting and appropriate.
Summary: In summary and as before, this Ethiopian study of IPV readiness with a large sample size using a well-respected tool, the PREMIS, has much to recommend it.
References: Correctly formatted.

7. PLOS authors have the option to publish the peer review history of their article ([what does this mean?](https://journals.plos.org/plosone/s/editorial-and-peer-review-process#loc-peer-review-history)). If published, this will include your full peer review and any attached files.


If you choose “no”, your identity will remain anonymous but your review may still be made public.


**Do you want your identity to be public for this peer review?** For information about this choice, including consent withdrawal, please see our [Privacy Policy](https://www.plos.org/privacy-policy).

Reviewer #1: No

Reviewer #2: No
